# Establishing research and translation priorities and care pathways for families where a parent lives with mental ill health

**DOI:** 10.1371/journal.pmen.0000266

**Published:** 2026-06-15

**Authors:** Darryl Maybery, Gavin Davidson, Anne Grant, Geneviève Piché, Scott Yates, Torleif Ruud, Addy Dunkley-Smith, Michelle Banfield, Jennifer Bibb, Dana Jazayeri, Victoria J. Palmer

**Affiliations:** 1 Department of Rural Health, Monash University, Melbourne, Victoria, Australia,‌‌; 2 The ALIVE National Centre for Mental Health Research Translation, Melbourne, Victoria, Australia; 3 School of Social Sciences, Education and Social Work, Queen’s University Belfast, Belfast, Northern Ireland; 4 Department of Psychoeducation and Psychology, University du Québec en Outaouais, Gaqtinea, Quebec, Canada; 5 School of Applied Social Sciences, De Montfort University, Leciester, United Kingdom; 6 Institute of Clinical Medicine, University of Oslo, Oslo, Norway; 7 Mental Health Services, Akershus University Hospital, Lørenskog, Norway; 8 Centre for Mental Health Research, Australian National University, Canberra, Australian Capital Territory, Australia; 9 Department of General Practice and Primary Care, University of Melbourne, Melbourne, Australia; PLOS: Public Library of Science, UNITED KINGDOM OF GREAT BRITAIN AND NORTHERN IRELAND

## Abstract

Service users and carers have recently had input into establishing mental health research and translation priorities in Australia. This study extends that work to include the voice of those with lived experience of parental mental ill health. Although, parental mental ill health is a global public health issue there is a substantial gap in knowing the research and implementation priorities for parents and their families. This study sought to establish the priorities that families with lived experience of parental mental ill health have for support and intervention, along with what social factors they identify as important to positive and negative outcomes. There were seventy-eight respondents to two open-ended survey questions related to mental health care improvements and social determinants impacting mental health. Respondents were parents with lived experience of mental ill health (n = 43), partners of parents (n = 11), family members (e.g., aunty, sibling; n = 14), and adult children of a parent (n = 10). Approximately 80% described their gender as female and 27 contributors reported having multiple lived experiences/roles (for example, as a parent and an adult child). More than 500 responses to the two open ended questions were entered into Leximancer 5 analysis software. The key priorities were: strengthening family member relationships; supporting children earlier and providing whole of family/group approaches; the need for practical, emotional and social elements of support within families and at home to address stress; increased well-being, and improved family cohesion and communication. Social priorities were access for childcare, community groups, therapy, and respite care, with an emphasis on relieving pressure points and promoting family cohesion. Intergenerational trauma and young caregivers were also highlighted, as were social determinants including reducing poverty and improving finances along with housing stability. These findings form priorities for future mental health research and have already led to the establishment of implementation actions for guiding future efforts by governments, service providers and researchers.

## Introduction

Establishing mental health research and translation priorities has commonly relied upon burden of disease data and/or the agenda of funding bodies, rather than being determined by the direct priorities of people with lived experience of mental ill health including carer, family and kinship groups [1,2]. Driving the mental health research and translation agenda from the perspectives of people with lived experience is essential to best focus service delivery, to develop associated policy and to conduct research that meets what matters for the people most impacted [2]. A critical challenge is that at least in an Australian context, mental health research and the services that are provided, appear to be disconnected from whose priorities are being addressed and how they are being met [1,3–5]. The case for a research and translation agenda shaped by services users and carer, family and kinship groups is becoming clearer [6]. Coupled with a clear need to monitor and report on progress in meeting priorities and in-depth consideration of how to identify the nuanced priorities and needs of families where a parent lives with a mental health challenge; which was the objective of this project.

Internationally, it is well recognised that parental mental health concerns can strongly impact families and children [7,8]. A major part of the problem is how families are considered and engaged by services, in that, “current practice paradigms are based on individualistic models of practice, particularly in mental health services” [9]. It means family/group and relational approaches to support lag. In research priority setting, people with lived experience of mental ill health (including carer, family and kinship groups) have had patchy involvement in developing and deciding on which research is conducted and what services may be delivered [10–13]. Where they have been engaged, it is unsurprising that amongst the most common service needs and reorientation has been this need for family focused practice [14]. The perspectives of parents and children affected by parental mental ill health have been explored to some extent in previous studies [15–18]. A recent priority setting study by Powell et al [15] interviewed parents with a history of depression and their children and identified five research priority categories: treatment and intervention; environmental and social factors; public understanding of mental-health; the role of genetics and intergenerational transmission and; a developmental and intergenerational approach to research. Other qualitative studies with parents with mental ill-health highlight the experiences of: a lack of support; difficulties in finding help; the need for balanced service approaches; the importance of effective promotion [16] as well as the desire for being recognised and supported for parenting roles and; the positive impacts of utilizing their service user expertise [17]. Qualitative studies of the experiences of children emphasise the importance of the children’s understanding of parental mental ill-health, parent-child relationships, coping strategies and social connections [18]. While priority setting exercises with children also exist these are not focused on the experience of having a parent with mental ill health [19,20]. Overall, while perspectives from parents and family members experiencing mental ill health exist, formal priority setting exercises are rare and limited in scope.

Children with a parent with mental health challenges are often considered ‘hidden’ because they often go unacknowledged and their needs unmet [21,22]. In adult mental health services in Northern Ireland, McCartan and colleagues recently asked the question *Are we starting to ‘think family ’yet?* and generally found limited evidence of a whole family approach in services [23]. Focusing on families is essential given that during childhood, an estimated 21–23 percent of all young people reported living with a parent with a mental health challenge [24]. According to Australian Institute of Health and Welfare data for 2017, approximately 16% of children age 0–14 years old co-resided with a parent where poor mental health had been reported [25]. A review of adult psychiatric service prevalence data internationally showed that across nine studies there were between 12–45 percent of attendees who were parents [26]. A 2013 national census of 23,167 outpatients attending 107 Norwegian adult mental health clinics found that 8035 (36%) were parents with children under 18 years [27]. Equally, a review of the literature showed that among young people attending child and adolescent psychiatric outpatient clinics in The Netherlands 36% reported having a mother with mental ill –health and 33% a father with mental ill-health [28,29]. Findings from the Youth Wellbeing Northern Ireland Survey also confirmed 23.8% of parents (N = 2816) reported experiencing a mental health challenge in the past and 15.8% said that they were currently experiencing challenges [30,31]. The international and national data is clear that parental mental ill health is common, but family, parental and child needs are not well responded to in current models of care with stigma, shame and individualised support impacting on this.

Having a parent with mental ill health can also have a substantial impact on young people and their families. Bunting and colleagues [30] found that there was an association between parent and child mental health. When looking at the issue from a symptom-based lens, children whose parents reached the cut-off score (4/12) on the General Health Questionnaire (GHQ-12) for probable mental health concerns, were twice as likely to meet the threshold for an anxiety or depressive disorder themselves. Children of parents receiving specialized psychiatric services were more likely to develop mental health challenges, with 34% of children experiencing mental health symptoms in what may be termed ‘the high-risk range’ [32]. In this context children were said to face challenges around school readiness, higher rates of physical injury, increased likelihood of being taken into out of home care, and heightened risk of developing physical health conditions such as asthma [7,33].

Along with the prevalence of parental mental ill health the data clearly shows that the impact on children can be vast and endures over the life course. The socio-economic and environmental influences on this play a crucial role in longer term outcomes for young people [7]. Notably, a review of family prevention and early intervention approaches found that they may reduce children’s likelihood of developing mental ill health by 40 percent [34]. Given that such prevention pathways can reduce mental ill health in adulthood [34], investment and directed effort is important.

The objective of this research was to establish the care and research priorities of parents living with mental ill health and to consider this in a family mental health context as part of the co-design of a living, national roadmap for mental health research translation [35]. The work was underpinned by the theoretical principles of family and personal recovery [36,37], including participatory research as related to the roadmap development and the concept mapping. The latter provided a planned approach to identifying priorities (see Christensen et al., 2013) with family members with lived experience of parental mental ill health. Theoretical principles of recovery were also central to the research, stressing family centred principles and care [38].

The priorities gathering exercise was a partnership between the Prato International (PaRent And child menTal health research cOllaborative; hereafter referred to as the Prato Collaborative) and Australia’s first mental health research translation centre (“the ALIVE National Centre”) funded by a special initiative in mental health by the National Health and Medical Research Council (GNT2002047). The Prato Collaborative was established in 2013 to advance the causes of young people and families where a parent has a mental health challenge – principally to make children and families more visible to health and social care services and other researchers [9]. The objectives of the ALIVE National Centre have been centred around addressing the priorities of the people most impacted for changing mental health research, and care delivery as articulated within the roadmap. The roadmap includes tailored pathways where priorities reflect the needs of specific communities such as Aboriginal and Torres Strait Islander people, younger people aged 16–25 years old and families where parent/s are living with mental ill health [39].

This study gathered the perspectives on priorities for research and care settings and specifically identified what would be the most successful approaches to working with families and the supports that could be provided to families where a parent experiences mental ill health, including what beneficial changes this would lead to from parent perspectives. It also examined the most important social determinants and issues to better understand the impact of relationships and contexts/environments on outcomes and experiences of families where a parent lives with mental ill health.

## Method

### Ethics approval

The ethical aspects of this study were approved by the Monash University Human Research Ethics Committee (MUHREC) (Project ID 24861). Consent was implied by choosing to complete the voluntary online survey. This was clearly stated in the introductory page of the survey as follows,

“choosing to enter into the online survey implies that you consent to participate. If you enter the survey, you can discontinue participating at any point before the point of submission by closing your web browser. Because this is an anonymous survey, it is not possible to withdraw your response after your survey has been submitted.”

Additionally, consent from minors (under the age of 16 years) was not warranted because the survey did not include people younger than 16 years of age nor were there questions that would make respondents of 16 and 17 years age “vulnerable through immaturity” as per the Australian National Statement of Ethical Conduct of Human Research 2023 (pg. 68, section 4.2.8).

### Survey instrument and recruitment

This study was carried out by an interdisciplinary team that included lived-experience researchers, with data collected via an anonymous Qualtrics survey. A link to the survey was shared to the ALIVE National Centre for Mental Health Research Translation networks, Prato member research networks, relevant Australian based mental health bodies (n = 162) and to specific external researchers via e-mail (n = 89) and also the wider community via social media. In addition to families and parents living with mental ill health, the survey was also distributed to other respondent groups (e.g., service providers) and across different countries for a separate purpose [40]. The recruitment period for the survey was from January 3^rd^- March 13^th^, 2023. People self-identified as either a parent, partner, family member or an adult child (16 years and above) whose parent/s live with mental ill health.

Survey questions were developed by the Prato Collaborative. For this analysis, the survey questions were grouped into the three priority areas of the 2022–2023 ALIVE National Centre’s national roadmap: mental health research, mental health care improvements and social determinants impacting on mental health and the determinants to address in new models of care. The mental health research findings are not included in this paper as they had a particular focus on co-design of measures which are being developed by the Prato Collaborative and reported separately. The mental health research priority topics still informed the co-design processes for establishing the pathways of families where parents are living with mental ill health, creating implementation actions that then are presented in Consensus Statements [41]. The mental health care improvement survey questions were:

- What are the most important things that families, where a parent has a mental illness, want to see change in their lives (from receiving services)?
- What should the purpose of interventions or supports for families be?
- What does it look like/what changes when an intervention is successful for a family?
- What does it look like/what changes when an intervention is NOT successful for a family?

The social determinants/social issues survey questions were:

- Should interventions include work to improve relationships within families? Why or why not? If so, which relationships should interventions seek to improve and in what way? e.g., relationships between family members, and/or with people outside the family.
- What characteristics of a family, an individual or their context/environment might affect the outcomes and experiences of families where a parent has a mental illness?

### Respondents

The 78 respondents identified as four lived experience groups across parents living with mental ill-health (n = 43), partner/s of a parent (n = 11), a family member (e.g., an aunty, uncle, sibling or grandparent) (n = 14) and adult children of a parent (n = 10). There was no pre-established sample size for the survey. The purpose was to develop theoretical rather than quantitative saturation following established qualitative designs [42] and no measures were included within the survey that were dependent on a specific sample size to determine an outcome [43]. Table 1 shows respondent characteristics according to self-selected lived experience groupings.

**Table 1:** Respondent age, gender, relationship status, reported diagnosis and education according to grouping.

Grouping (N;%)	Age * (mean, SD)	Gender	Relationship Y or N	Mental ill health Y or N **	Reported Diagnosis	Highest level of education***
Parent (n = 43; 55%)	45.28, 10.31	42 Female 1 Fluid	32 Y 11 N	43 Y	24 Depression 23 Anxiety 10 Post Traumatic Stress Disorder 5 Schizophrenia 10 Other	14 Postgraduate, 14 Undergraduate, 11 Trade/Diploma, 4 Secondary
Family member (n = 14; 18%)	41.50, 14.03	12 Female 1 Male 1 Non Binary	8 Y 6 N	6 Y 7 N	2 Depression 2 Anxiety 1 Post Traumatic Stress Disorder 2 Other	7 Postgraduate, 3 Undergraduate, 3 Trade/Diploma, 1 Secondary
Partner (n = 11; 14%)	41.55, 7.54	8 Female 3 Male	10 Y 1 N	5 Y 6 N	3 Depression, 4 Anxiety, 1 Post Traumatic Stress Disorder	4 Postgraduate 5 Undergraduate 1 Trade/Diploma 1 Secondary
Adult children (n = 10; 13%)	26.80, 10.39*	6 Female 2 Male 2 Non Binary	7 Y 3 N	10 Y	8 Depression 7 Anxiety 1 Post Traumatic Stress Disorder 4 Other	1 Postgraduate 4 Undergraduate 5 Secondary

* Note that one respondent was 50 years of age – the average age of this subgroup without this respondent was 24.22 years.

** Numbers do not all equal the whole sample.

*** Postgraduate: further study after completing a Bachelor’s degree; Undergraduate: Bachelor’s degree; Trade/Diploma: Vocational training, technical school or certificate Secondary: High school.

The majority (55%) of the survey respondents were parents and the remaining three groups were relatively evenly distributed respondents with adult child of a parent (n = 10), partner (n = 11) and other family member (n = 14). Approximately 80% of the overall sample, and 70% of those who were tertiary educated (for example, university), identified as female for their gender descriptions. Respondents also identified if they had other lived experience perspectives in addition to the one that they indicated as their primary perspective for inclusion in the study. This resulted in a total of 111 distinctions across different lived experience perspectives. For example, 21 contributors reported sharing two perspectives and six indicated that they were sharing three lived experience perspectives in response to survey questions. These dual and multiple lived experience perspectives were commonly overlapping with family member, parent and/or partner roles, with only one respondent (other than the adult children indicated above) suggesting that they had also experienced being a child from a family with a parent with a mental health challenge. Notably all respondents who had grown up with a parent with mental ill health self-identified as experiencing mental ill health themselves.

### Data analysis using leximancer

Leximancer 5 is a desktop software analytics program that uses advanced algorithms to identify key concepts, and their relationships within given text [44]. It is an efficient alternative to manual coding with high concordance to manual coding analysis [45]. Leximancer is a computer-based software tool to support qualitative analysis. The program identifies the co-occurrence of semantic and relational concepts within text [46] and presents these as networks within themes as a map. Leximancer was used to analyse the grouped responses to the open-ended survey questions in each priority area of the roadmap: mental health care improvements and social determinants/social issues. The concepts that were identified formed the priorities that subsequently guided the public co-design of implementation actions for the Consensus Statements (called Pathways of Families) which provide directions on actions that can be undertaken within research, policy and practice [41]. Leximancer analyses the text-based answers provided by people and presents these as a visual map of concepts positioned within larger themes. Concepts appear as individual nodes on the map either connected to other concepts that are discussed in relation to these or as connected concepts. All maps are linked with the textual responses for further analysis. A concept is identified by a cluster of commonly co-occurring words (or put another way, words that travel together) within the text. The concepts are organised relationally and then grouped by Leximancer into themes. The concepts are understood as either relational or interconnected which is represented by grey lines that show these connections on the map that is generated from the analysis. Concept circles vary in size depending on the prominence within the textual responses. Sometimes, a concept can share its name with a theme (e.g., ‘support’), which reflects both the data’s conceptual content and the thematic structure suggested by Leximancer. The spatial arrangement and size of the circles in the concept maps represent the frequency and prominence of each concept in the text. It is important to note that the Leximancer analysis is not solely based on frequency of the concept, but that the relationship also is in focus.

The Leximancer generated maps use a colour scheme or heat map approach. This means colours such as red denote the most frequently discussed concepts, progressing through shades of the colour spectrum (e.g., brown, orange, green, blue, to purple) to represent the less frequently used concepts. The analytical process involves several stages, starting with the upload of an initial .csv file into Leximancer, followed by a preliminary analysis to produce an initial map. This map displays the primary themes and their interconnected concepts, and it helps to identify overly prominent concepts and common filler words, referred to as stopping words (e.g., “think,” “maybe”). These are typically removed in subsequent steps using Leximancer’s concept seed editor function. Further iterations refine the map by merging singular and plural forms of concepts. Following this, map iterations are completed to arrive at the final stage map. At this stage common words that are used within survey or interview questions are also removed as these tend to be repeated in people’s responses also.

The final map is then used in conjunction with textual analysis to further examine the meanings suggested by people about the generated concepts and the interrelationships of these. In this study, the examination of the concepts and text was to establish what the priorities for mental health care improvements and social determinants were for parents living with mental ill health, their partners, family members and adult children.

Leximancer is a robust text analytics platform [45,46]. However, the researcher is responsible for providing the final interpretation and description of the themes and concepts to explain the interconnections and size of the mapped output. The software provides the map of the themes and concepts, but the researcher engages in the examination of the meanings of these with the text that is associated with the map. The origin of verbatim comments is acknowledged in the findings according to the lived expertise identified (e.g., partner, family-member or adult child), gender and age.

### Findings

The following presents what 78 respondents said mattered in the priority areas of mental health care improvement and social determinants from the ALIVE National Centre and Prato Collaborative annual lived experience priorities survey conducted in 2023.

### Mental health care improvements

**Fig 1:**
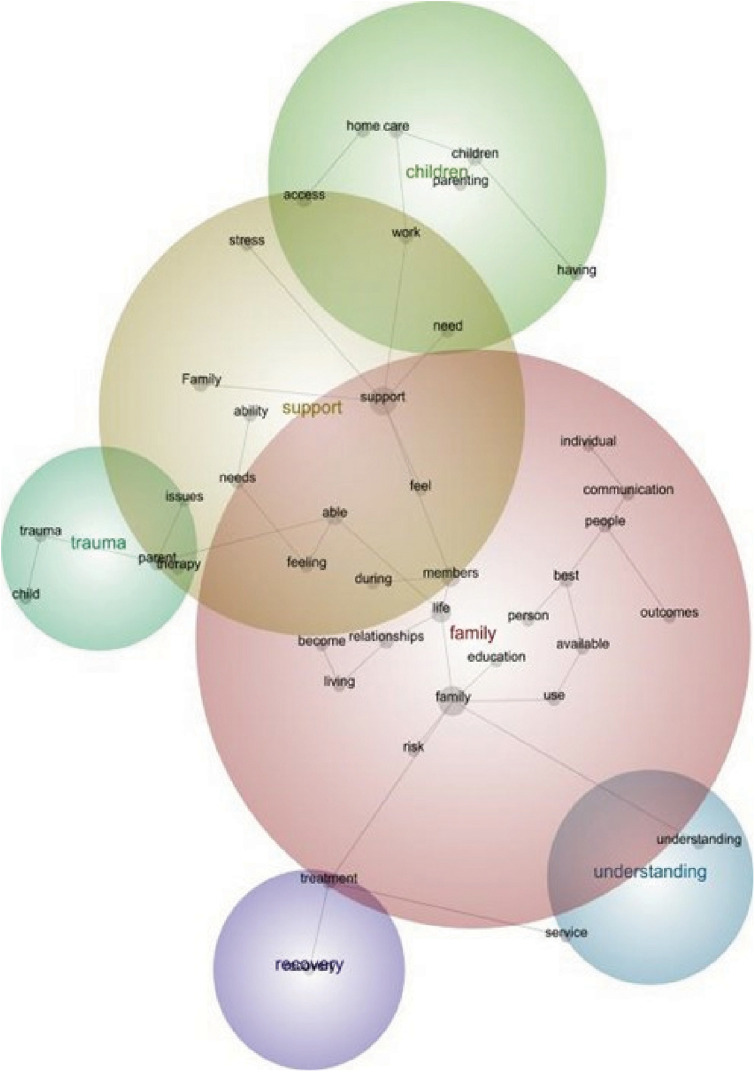
Leximancer concept map for responses to mental health care improvements questions‌‌.

Fig 1 shows six conceptual groupings by Leximancer into the main themes of the important concepts for all respondents. The strongest theme was “family” which is reflected in both the size (very large) and colour (red) of the thematic circle and overlaps with themes of “support” (brown/yellow), “children” (lighter green), “trauma” (darker green), “understanding” (blue) and “recovery” (purple). Concepts within the themes are shown by grey dots within these circles and the grey connected lines between concepts illustrate the relational or connected nature of them. For example, from the core concept of family, there are interrelated and connected concepts such as between family and education, or between people, communications and outcomes. Other conceptual connections are shown between the concept of family with risk, and the concept of family with treatment (also connected with service in the blue circle of understanding) which connects to recovery. Final conceptual relationships link family with understanding and services overall.

Core to people’s priorities illustrated in the Fig 1 map, was the concept of support which is represented by the yellow brown thematic circle. Support as a concept showed strong connections again with family (hence the thematic circles show significant overlap). This reinforced that people’s priorities were about the need for support to focus on and engage with families. The connection between support and stress also showed that these priorities were independent of others and might be interpreted as stress being evoked where support is not provided. This is illustrated in the response of a 49-year-old female parent, who shared that “This is very individual. Some people may want to gain confidence in their parenting skills. Others may need access to financial support or to deal with other psycho-social stressors”. Support that is driven by families, then, should be a key focus of interventions and the extent to which it is perceived as being in place may be the measure of success. Respondents suggested that the best support needs to be delivered to the person in a way that takes account of family member’s needs and “all members of the family have the capacity and support to live the life that they want” (Parent, Female, 62).

The relationship between support for direct needs and for work also moves into the theme of children (represented by the green thematic circle). The grey connected lines help to understand the connections between concepts further. Here, we can see that families see support for work, care and home and access to these supports as a priority. The theme of trauma is also overlapping with support. Therapy has been discussed as a prominent concept, but it is positioned on the edges with parents and interrelated with trauma and child as priorities to be addressed. This was highlighted by the response, “…enhance protective factors and explain treatment/therapy/support options for all in the family” (Parent, Female, 38) and detailed as follows, “Family support - home help, fully funded childcare, local community groups - mothers’ groups, playgroups, access to affordable and coordinated clinical and psychosocial support services. Regular quality General Practitioner (GP)” (Parent, Female, 42).

For parents, partners, family members and adult children, these concepts reflect core priorities for mental health care improvements. For these improvements to be successful, parents suggest that “The family can confidently look after the person with a mental illness without feeling overwhelmed, not knowing what to do, or where to look for help. I know my husband felt very lost and didn’t know how to support me.” (Parent, Female, 42).

Respondents indicated that this support should have the characteristics of being “non-judgemental (e.g., Partner, Female), practical and emotional and socially oriented, with more support in schools for kids” (e.g., Parent, Female, 26) and support to parents and partners from General Practitioners. Overall, parents and their families want support that is *kind* and *inclusive* so that they can trust service providers. Family members need support in their own right and experience “burn out from supporting their loved ones” as well.

Parents living with mental health problems also want information provided to their partners for what is happening. This is reflected by education appearing in the family thematic circle as a prominent concept, “where can they [families] get help and how do they support a person with depression?” (Parent, Female, 42). Another respondent suggested that “active support such as good, ongoing information about my own and my children’s conditions and different treatment possibilities is good” (Parent, Female, 63 years). “Access to services to relieve the pressure points (cleaning, meals, transport to appointments)” (Parent, Female, 63 years).

The need for practical support, as highlighted in the previous quote, was reinforced by many respondents and “respite care for those who are full on single solo parents without support or a co-parent” (Parent, Female, 26) was seen as a core area of need. For parents living with mental ill health, “being able to carry out the daily tasks required at home and care for your family and being able to work” (Parent, Female, 37) are priorities. This support, however, should be extended to partners, so that “daily living support is available when a parent is hospitalised for treatment.” (Parent, Female, 46).

However, some respondents highlighted that “when I don’t have access to supports that recognise full-time work commitments, parenting and other commitments, it sends the message that I am not bad enough yet” (Parent, Female, 42). Such priorities around the concept of support illustrate the importance of education within for families and access to preventive models of care that reflect the supports being outlined.

Key to the priorities for families are unsurprisingly children and their wellbeing. For some parents, there was a view that “children may be neglected and children [may] run the family as a young carer for their parent.” (Parent, Female, 39). This facilitated some parents sharing worry about the risks to children for future wellbeing and mental ill health. Others suggested that they want “family centred support and access to trauma therapy for parent issues so that they [the trauma issues] don’t extend to the kids” (Parent, Female, 42). Parents want mental health care “to help families cope with what they find most difficult or overwhelming so they can function to care for children and ultimately themselves” (Parent, Female, 34). There was a sense that more, “thorough support services and for doctors practitioners and psychologists to take sleep, diet, exercise, and other mindfulness activities more seriously. [I] found that enough questions were not asked to support my recovery and I was left to figure a lot out by myself.” (Parent, Female, 34).

Parents also wanted “to keep children thriving and feel safe and secure and know that they are loved and cared for by me” (Parent, Female, 63). The kinds of risks that parents want to be reduced are those to “keep [the] family together [and] reduce risk of intergenerational trauma by working with kids and parent[s] together and separately.” (Parent, Female, 39). In this regard, whole of family interventions and centring collective approaches reflect the priorities for people with lived experience of mental ill health. Parents see this as: “ensuring that all family members live the life they want, [are] support[ed] to be the best person that they can. Ensuring that information, techniques, tips and skills are available for people to learn so they can use them to understand and live the life they want to live. So that all members of the family thrive not just survive” (Parent, Female, 62).

For family-oriented care provision, the key concepts are understanding, relationships and communication. These point to specific processes and outcomes in care that matter for families. Parents expressed worries that current approaches overlook the needs of partners and/or children and other family members, and that many children living with a parent with mental ill health may also have mental health needs. Based on the responses these needs remain unmet.

Those outcomes are also connected with the kinds of interventions (educational) and treatments (recovery/trauma) that are shown to be important to offer as shown by the grey line connecting these concepts across the map in Fig 1. To establish family-centred outcomes parents suggested that we need to look for “when the whole family benefits,” “when the family can stay together”, ‘age-appropriate measures for identifying when things work’, “communication improving between family members”, and how “everyone feels safer knowing how to move forward. Family members are happy and able to enjoy daily activities as an individual and as a family.” (Parent, Female, 41). Other indicators that care provision is going well included that the person can “enjoy life and participate in family life and work.” (Parent, Female, 37). Most importantly, parents’ priorities are for the family to define what is needed, successful or going well in care.

When services and interventions are not successful, parents suggest that the outcomes include, “suicide attempts, entrenched domestic family violence, domestic abuse, unhealthy addictions, unhealthy relationships, unhealthy behaviours, poor quality of life.” (Parent, Female, 42). For mothers the sense is, “in my case, as the mother, if I’m down then the whole family suffers. I manage the mental load and life admin for everyone.” (Parent, Female, 32). When care provision is not working, parents feel like, “family members are encouraged to act as pseudo-clinicians and take responsibility for ensuring compliance with bio-medically focused treatments which increases conflict within family and the family trust/relationships are destroyed.” (Parent, Fluid, 56).

These areas for mental health care improvement also intersect with priorities for families about social determinants and social issues that impact on the experiences of living with mental ill health as a parent. These are explained in the next section and point to further areas for focus within new models of care for implementation and re-designed or enhanced service delivery. These are critical considerations with the pathways for families in the ALIVE National Centre’s roadmap.

### Social issues and determinants

**Fig 2:**
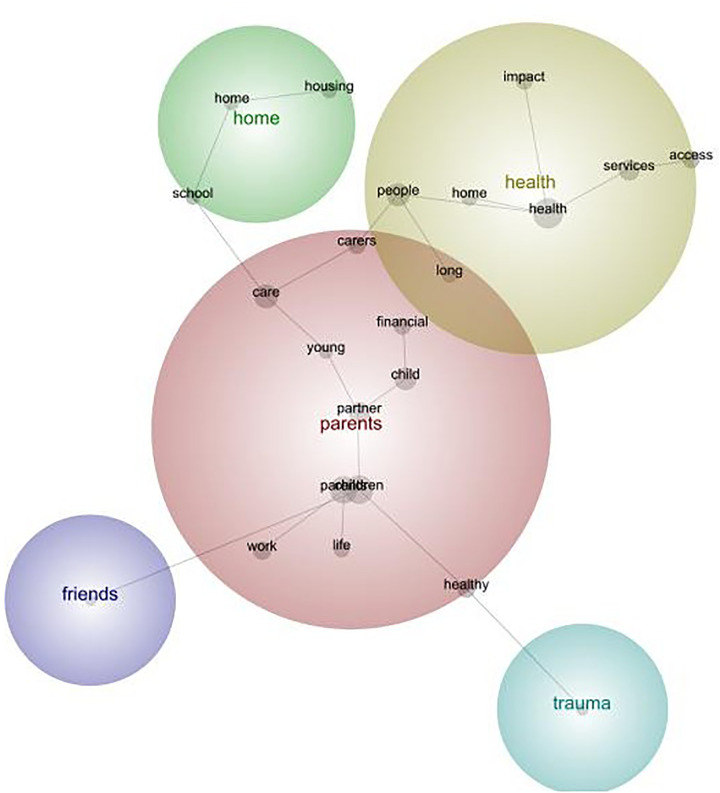
Leximancer concept map for responses to social issues and determinants.

Fig 2 illustrates how social determinants and relationships are central to the priorities of families in the models of care or supports that they receive. In the main theme parent (illustrated by the large red thematic circle), the connection between the concepts of work and finance were made clear as priorities that are linked with life and needs. This can be understood further by one parent’s response which suggested that “work helps with the social needs of parents and healthy separation from children, [and] living a productive life with good role modelling for children building structure.” (Parent, Female, 31). Work similarly therefore is a priority for parents because it can be a contributor to being healthy (shown by the brown/gold theme) and a need for supporting families. Alongside, services and access play a role in familial wellbeing too. The connections between grey lines also indicate that partners and carers and other people play a critical role in wellbeing and support. Friends were also noted as important to include in care and services too, for example, “[it] shouldn’t just be biological families, chosen families (close friends) should be included too” (Adult child, Female, 25).

Different perspectives on the role of the home emerged for respondents from culturally diverse backgrounds. Here, a respondent raised the issue that if there was manipulation to stay at home this could be a problem. Another respondent suggested that factors such as “lack of transportation and insecure housing” (Adult child, Female, 25) contribute to a lack of wellbeing and, from another point of view, core priorities linked with social determinants were: “openness to learn, economic/ housing stability. If I can only focus on where the next meal is coming from or where we are going to sleep tonight - non-immediate things like relationship restoration/strengthening will not gain my attention, focus or priorities.” (Family member, Female, 62). Housing and community were priorities for respondents as well because of “self-stigma and previous negative experiences with government and social workers” (Family Member, Male, 30). Stable housing was raised several times by respondents as a major contributor to wellbeing.

Poverty was seen by one adult who identified as growing up with a parent living with mental ill health, “to be the most relevant [because] poverty and lack of resources in a family can magnify small problems, right?” (Adult child, Male, 50). For one adult child who shared their perspectives based on growing up with a parent with mental ill health, they said, “One of the most significant issues that I experienced were poverty. Financial barriers - exaggerated by things like location, drug and alcohol use/access, various systemic prejudices, and bureaucratic systems that actively disadvantage those who are mentally, neurodevelopmentally or neurologically disordered - are one of the hardest to overcome when it comes to consistently accessing treatment. It is also the characteristic which has provided the most additional stress outside of the mental illness itself, in my opinion…The internal dynamics of a family will also affect the outcomes produced. In my experience things like prejudice within the extended family, co-dependency, enabling, and parentification were the things that had the most negative impact. On the other hand, complete separation did not help at all, either, because my mother’s support needs were too high and her mental health nose-dived in a number of ways when it occurred.” (Adult child, Non-binary, 22).

For some respondents, the reason the parent-child relationship should be at the centre of priorities is because it is “the most common caring relationship” (Family member, Female, 47). For family members, parent child relationships should be centrally in focus because, “child removal practices currently are not distinguishing between places where there might be irredeemable harm and places where parents could do a “much better job if they had support” (Family Member, Nonbinary, 32).

Finally, trauma was not as prominent a concept as the determinants of health, housing and home life, and friends but it remained a priority for implementation within supportive models of care. People also suggested that more than simply a trauma-informed approach is needed with an emphasis for family relationships being improved by “relational trauma recovery is needed” (Parent, Female, 42).

## Discussion

The objective of this study was to expand the knowledge base of the perspectives of those with lived experience of parental mental ill health on what matters for mental health care improvements and the social determinants that are important for models of care to be implemented for system redesign Following this, implementation actions were co-designed and presented within the ALIVE National Centre’s roadmap for mental health research translation. These actions were represented in the Phase 2 Consensus Statements which are readily available online and have been opened more than 42, 000 times internationally.

This first aim of this study was to determine what service approaches and supports should be provided to families when a parent experiences a mental health problem. The findings highlighted that: support for the whole family, and early support to children within families and to apply social and practical solutions to increase family cohesion and communication mattered. These findings are not dissimilar to what was shared in publications related to developing research and service priorities with families and children where a parent lives with mental ill health [15–17]. Regarding social determinants, services, research and policy should target factors that reduce stress and pressure for the family (e.g., respite, childcare), offer practical assistance such as housing and financial security and address being a young carer and the impacts of ongoing family trauma.

In many respects it is unsurprising that respondents focused on strengthening family member relationships and the need for support within families, given people are speaking as parents and care givers largely. But the fact that these are repeated priorities has confirmed continued gaps in care delivery to address the whole of family needs and to attend to social determinants within models of care. Also noted was the importance of family as a support and for making meaning of experiences compared with the individualised symptomology or illness severity of the unwell parent. This reinforces the importance of relationships in addressing stress and ensuring familial wellbeing and widens the lens for a collective view on supports and care. Practical, emotional and social elements of support were illustrated for different ages and groups of family members and these priorities centred around the home environment and its role in healthy outcomes. Parents emphasised the need for non-judgmental, inclusive support that acknowledges individual circumstances and challenges but that works holistically. For many, person centric systems of care and service models continue to overlook the family system and collective approaches. The focus on direct individual care provision ignores the needs of family members and the priorities for parents around preventing future impacts of their own health on children. The latter is a common concern raised by parents living with mental ill-health.

Key support priorities that parents, partners, family members and adult children of a parent shared included access to childcare, community groups, therapy, and respite care, practical support in the home with an emphasis on relieving pressure points and promoting family cohesion. Concerns were raised by parents living with mental health challenges about neglecting children’s needs and intergenerational trauma, which underscore the importance of family-centered care and collectively or group-oriented interventions where families are not biologically related or may be comprised of supportive friends or other caregivers. Successful models of care for family members were defined by being able to experience improved communication, feel understood, and the ability for all family members to thrive, this included an emphasis on families as the drivers of wellbeing or good support themselves. Conversely, when asked about when support or a model of care does fail, families suggested that this was experienced as continuing to face significant challenges such as domestic violence, addiction, and strained relationships and importantly it included the threat of child removal and placement of children into out of home care. Overall, there is a call from this group for comprehensive mental health care improvements that encompass family dynamics, a focus on relationships with practical support and social determinants that provide holistic support in systems aligned with the diverse needs of families.

In terms of improving within family relationships, respondents particularly highlighted the need to improve understanding and communication between parents and children. This has been a key focus of previous interventions that have been found to reduce the impact of the parent’s ill health on children [34] and often these interventions have focused upon improving parenting functions and the relationship with and knowledge of the parent’s problems with children. A review by Siegenthaler et al., [34] included an intervention called Let’s Talk about Children that has been shown to improve outcomes over the longer time frame for both parents and children [47]. However, these approaches generally only engage with the parent, fewer jointly target both children and parents and aim to improve relationships as a holistic approach [48]. As outlined in the introduction to this paper a systemic weakness in current mental health care provision is that children are often not recognised within models of care [21] and ‘family thinking’ is rare in adult services [23] and it is rarer still in program planning and co-design of models of care. Efforts have been made to encourage mental health service providers to initiate conversations with the parents in their care about their parenting and to work on ways to improve their relationships with their children where needed [49,50]. However, adult services often do not consider children’s needs within the scope of services [24,51] and have variability in their skills and confidence to work with children [12,52,53].

The findings of these priorities and the analysis also highlight that partners, carers, and friends are also recognized as important relationships to be enhanced, emphasizing the need for inclusive support networks and consideration of the relational and cultural determinants in new models of care. Previous research has illustrated the role of other family members including the parent’s intimate relationships [54] and redistribution of family roles due to the parent’s ill health [55] along with the parent’s level of interpersonal and practical supports (e.g., home-based service support [55]). For young people, social support [56], relationships generally [57], social skills [55] and parent-child interactions [58] have all been the focus of past research. Relationships have been regularly measured in past research with these groups [48,59].

Regarding young carers, a recent comprehensive scoping review has highlighted limited quantitative research and few young carer support programs [60]. Only two studies focused upon young people and children in a carer role with the parent and/or other family members [48,55,56]. In Norway, Kallander and colleagues [55] found that a young carer’s quality of life was negatively impact by their caring role and Mechling (2015) [56] has suggested both positive and negative, albeit modest, relationships between caring and wellbeing. Other research has suggested caring responsibilities and the roles of close family support to be a key focus for future parental mental ill-health research [9,48]. Caregiving is a worthy area for further investigation particularly for the implementation of earlier intervention and community level prevention.

Other key social determinants that are known to be very important and that were highlighted here are finances, employment, housing and poverty including the intersection of trauma within these. Trauma, along with the term stress [56], has received some recognition in past commentaries in the literature [9] particularly about child trauma [61], however our recent reviews of qualitative [59] and quantitative literatures [48] show trauma to be under researched with the few previous studies showing diverse findings and fewer still exploring the intersections of trauma-violence. Nordh et al. [32] found accumulated trauma impacting on children’s wellbeing and Garosi et al. [62] found parental mental ill health gender differences (not found with mothers) impacting on children. In the qualitative literature, Isobel et al. [63] takes this a step further by suggesting:

*Further research is indicated to clarify the concept of intergenerational trauma, including its relationship to transgenerational mental health and illness, alongside examination of ‘what works’ in intervention* (p. 639).

Parent wellbeing was another important area for focus and is an area that could be included in future iterations of the ALIVE National Centre’s roadmap for mental health research translation. Many studies of parent mental health have focused on illness [48] and only one paper in the literature has measured quality of life [64]. The failure to acknowledge parental wellbeing is a major issue, particularly considering the emergence of the concept of personal recovery [36] and positive psychology [65] and need for prevention in the literature. While this should be included in the roadmap and pathways for parents, interestingly wellbeing has received considerable research attention in children of parents with a mental health challenge. For children the positive dimension has been extensively covered measuring such things as hope [66], life satisfaction [67], self-esteem [57], maturity [68,69] and confidence [70]. Alternatively, parent wellbeing, being measured on just one occasion, is a serious research shortcoming [48]. We must grow longitudinal and over time understandings within a relational approach.

Parents were, in this study, concerned about the impacts on children and prioritised the importance of trauma-informed approaches that can encompass a recovery and healing centred view. To some extent, they saw this as a more than current trauma informed care and the emphasis from respondents was to protect children’s future well-being and to ensure that support is delivered holistically using a family or systems approach. The concepts shared in responses highlight important nuances for pathways of family’s mental health care improvements and in social determinants to inform new models of care. This means that research activities and priorities need to prioritise these aspects and pay attention to the distinctive differences that may be important in Aboriginal and Torres Strait Islander family contexts, and across different cultural groups.

Many responses from parents in this survey expressed undertones about the future risks for their children. This concern was both in the context of intergenerational mental ill health that could develop, but also for carrying intergenerational trauma. In previous iterations of the co-designed roadmap, trauma has been one of the main priorities established in early work in 2022 followed by the need to review the deficit lens of intergenerational trauma and consider the issue from a perspective of compound trauma [35]. These worries need to be responded to within new models of care that are currently being implemented as part of the Australian government’s Medicare Mental Health Centres for kids and adults (formerly referred to as Head to Health). The priorities additionally, as we have noted, must be nuanced within particular groups with unique lived-realities and perspectives to share. The priorities established in this analysis confirm the needs for preventive systemic approaches that are embedded within whole of community models across the life course with child-centred approaches. In terms of priorities for research and how these are established, co-design approaches and involving families were key.

These findings extend those of the 2022 lived experience priorities national survey, which demonstrated the centrality of people’s experiences for mental health research and translation [3]. Similar to the current findings, the 2022 respondents described interconnected concepts of family, recovery, trauma and impacts, but these were positioned within a Leximancer thematic map that also had a strong focus on treatment and services over social determinants [3]. These differences may be partially due to the design of the studies, with the 2022 study asking a very general question about priorities for research whereas the current study asked more detailed questions about interventions and measurements consistent with previous families work and the Prato Collaborative aims. However, the differences also highlight the importance of focused priority-setting with specific groups and diverse lived experiences, to ensure nuanced priorities are captured [1].

### The pathways for inclusion in the ALIVE National Roadmap

As a direct result of establishing the priorities of parents living with mental ill health, priorities were reviewed in co-design meetings and implementation actions were developed for Phase 2 Consensus Statements for the pathways for families particularly for mental health care improvements and social determinants [41]. These areas were chosen based on a combination of the priorities for families from both the ALIVE National Centre and Prato Collaborative survey and the ALIVE National Roadmap’s existing priorities identified by families.

- Mental Health Literacy
- Young Caregivers
- Intergenerational Trauma
- Within family factors (wellbeing, relationships, communication)
- Social determinants, environmental and external factors
- Service Models/Configurations for Family Engagement (e.g., family-professional alliance, services family focus/support)

The findings from this study of people with lived experience of parental mental health challenges are important as they add substantially to the ALIVE National Centre’s roadmap and ensure that pathways for family’s priorities are established with co-designed implementation actions. These implementation actions are practical steps outlined in the publicly available Consensus Statements that provide guidance for researchers, policy makers and service providers to inform future efforts [41]. For researchers, they can help shape funding proposals or research projects, for policymakers, policy development and for service providers service design or improvement.

Importantly the priorities provide input to mental health research, improvements to care and signal the social determinants and issues that need to be addressed from the perspectives of the people most impacted. It is clear for parents who are living with mental ill health that a family/collective and relational focus is critical to future service delivery, associated policy and future research to meet the priorities of people with lived experience of parental mental ill health.

### Limitations

Respondents identifying as female (80%) and those who were tertiary educated (70%) were overrepresented in the current sample - demographic diversity is encouraged for future iterations of pathways and roadmap priorities. While a considerable proportion of the respondents identified with multiple lived experiences (noting that over 111 lived experience perspectives were shared) future research could examine for differences of opinion from lived experience subgroups (e.g., parents versus children). This survey did not cover the views of Aboriginal and/or Torres Strait Islander people where more work is planned to understand priorities for improving families support and care in the National Centre as an individual pathway in the roadmap.

## Conclusion

Effectively including and supporting whole families approaches (and collective models) in care is an under-addressed and under-funded area.. Findings from this annual lived experience priorities survey highlight clear areas for improvement and responses needed to address social determinants of health for future research and translation. The implementation actions developed from these priorities have the potential to considerably improve the delivery of services for parents, children and families as they interact with the mental health system. The pathways and what parents have prioritised also seek to bring the needs of children into plain sight rather than remaining hidden and unarticulated. Together these conclusions confirm the next essential step within the ALIVE National Centre’s roadmap for mental health research translation; to develop the pathways of children and young people (16–25 years) and ensure these priorities are not only reflected within mental health research translation efforts but responded to directly by researchers working in partnership with communities and families.
